# Potential of the Novel Slot Blot Method with a PVDF Membrane for Protein Identification and Quantification in Kampo Medicines

**DOI:** 10.3390/membranes13120896

**Published:** 2023-12-01

**Authors:** Takanobu Takata, Togen Masauji, Yoshiharu Motoo

**Affiliations:** 1 Division of Molecular and Genetic Biology, Department of Life Science, Medical Research Institute, Kanazawa Medical University, Uchinada 920-0293, Ishikawa, Japan; 2 Department of Pharmacy, Kanazawa Medical University Hospital, Uchinada 920-0293, Ishikawa, Japan; masauji@kanazawa-med.ac.jp; 3 Department of Internal Medicine, Fukui Saiseikai Hospital, Wadanakacho 918-8503, Fukui, Japan

**Keywords:** Kampo medicines, proteins, membrane chromatography, polyvinylidene difluoride membrane, slot blot, tris-(hydroxymethyl)-aminomethane, urea, thiourea, 3-[3-(cholamidopropyl)-dimethylammonio]-1-propanesulfonate, advanced glycation end products

## Abstract

Kampo is a Japanese traditional medicine modified from traditional Chinese medicine. Kampo medicines contain various traditional crude drugs with unknown compositions due to the presence of low-molecular-weight compounds and proteins. However, the proteins are generally rare and extracted with high-polarity solvents such as water, making their identification and quantification difficult. To develop methods for identifying and quantifying the proteins in Kampo medicines, in the current study we employ previous technology (e.g., column chromatography, electrophoresis, and membrane chromatography), focusing on membrane chromatography with a polyvinylidene difluoride (PVDF) membrane. Moreover, we consider slot blot analysis based on the principle of membrane chromatography, which is beneficial for analyzing the proteins in Kampo medicines as the volume of the samples is not limited. In this article, we assess a novel slot blot method developed in 2017 and using a PVDF membrane and special lysis buffer to quantify advanced glycation end products-modified proteins against other slot blots. We consider our slot blot analysis superior for identifying and quantifying proteins in Kampo medicines compared with other methods as the data obtained with our novel slot blot can be shown with both error bars and the statistically significant difference, and our operation step is simpler than those of other methods.

## 1. Introduction

Kampo medicine is a Japanese traditional medicine modified and developed based on traditional Chinese medicine from the fifth to the nineteenth centuries [1,2,3]. Kampo medicines were carefully selected and developed to include various crude drugs (natural products). The traditional Japanese formulation of Kampo remedies influences their selection [2]. Modern Kampo medicines have been produced from extracts using manufacturing methods governed by several national laws in Japan since the late twentieth century [3,4]. They are officially recognized and stipulated in the Japanese Pharmacopoeia, and their quality must comply with legal provisions [3,4]. Considering that the names of Kampo medicines are spelled in Chinese characters and pronounced in Japanese, Japanese researchers have organized the Standards of Reporting Kampo Products (STORK) to assign English names to Kampo medicines [5].

Randomized controlled trials of Kampo medicines have been performed to investigate their clinical effects [6,7]. According to Japanese industry, academia, and government, applying Kampo medicines for cancer supportive care is the goal for the twenty-first century [8,9,10]. Despite considerable efforts, the characterization of Kampo medicines’ components remains incomplete [11,12,13]. Analysis of Kampo medicine compounds such as Goshajinkigan and Ninjin’yoeito using three-dimensional high-performance liquid chromatography (3D-HPLC) has detected major low-molecular-weight compounds from the extracts [14,15,16]. For example, Jin et al. reported the clinical effects of each major low-molecular-weight compound for 34 crude drugs [17]. However, proteins have a high molecular weight. They must be extracted with high-polarity solvents, such as water and 70% ethanol aqueous solution (ethanol: water = 7:3) [18,19], making their solvent removal (evaporation and sublimation) and collection difficult. Moreover, given their relatively suitable concentrations, the proteins in cells or tissue lysates can be readily quantified using the Bradford and bicinchoninic acid (BCA) assays [20,21]. However, these assays may not be appropriate for quantifying protein concentrations in crude drugs. Accordingly, we evaluated the applicability and suitability of conventional technologies for the separation, detection, identification, and quantification of proteins in Kampo medicines [22,23,24]. We found that, given the insufficient crude sample volume, column chromatography [22,25], electrophoresis [22,26], and enzyme-linked immunosorbent assay (ELISA) [27,28] may be unsuitable for detecting rare proteins within the extract in Kampo medicines. Hence, we focused on membrane chromatography, where samples can repeatedly flow against the membranes, facilitating the collection and separation of proteins [22,23,24]. Although the membrane material varies and includes cellulose acetate, chitin, chitosan, nylon, and polyvinylidene difluoride (PVDF) [24], we consider that PVDF membrane chromatography is suitable for collecting proteins in Kampo medicines as the PVDF polymer is a strong, semi-crystalline material used in myriad medical instruments (e.g., surgical instruments) [29,30,31,32]. PVDF membranes also boast good membrane-forming properties, thermal stability, chemical stability, and mechanical properties [33,34]. Considering that PVDF membranes are often used as filters to produce clean water, natural organic compounds in wastewater, such as proteins and oil, were selected for removal [35,36]. PVDF membranes have also been adopted with the filter blot method for atmospheric particle matter proteins [37] and for electrospray ionization mass spectrometry (ESI-MS) analysis combined with sodium dodecyl sulfate (SDS)–polyacrylamide gel electrophoresis (PAGE) (SDS-PAGE) and “on-PVDF membrane digestion” [38]. In contrast, we focused on slot blot analysis as it is based on the principle of membrane chromatography; however, it requires only a simple and rapid protocol.

In 2017, we developed a novel slot blot analysis and quantified one type of glyceraldehyde-derived advanced glycation end product (AGE): GA-AGEs [39,40]. This method comprises a PVDF membrane and a special lysis buffer for the cell/tissue lysate [39,40]. AGEs are modified proteins formed by interacting with saccharides (e.g., glucose and fructose), their intermediate metabolites/derivatives, and protein [41,42,43]. PVDF membranes are commonly used to probe proteins in cells or tissues for Western blotting or slot blot analysis [39,40]. Herein, we hypothesized that the novel slot blot method for quantifying AGE-modified proteins could also be used to identify and quantify proteins in Kampo medicines as they share properties with other modified proteins, such as methylated [44,45,46], acetylated [47,48,49], phosphorylated [50,51,52], glycosylated [53,54,55], and myristoylated [56,57,58] proteins.

In this article, we compare the performance of our novel slot blot with other commonly used technologies (e.g., column chromatography and electrophoresis) and the other slot blot assay to assess its potential for identifying and quantifying proteins in Kampo medicines.

## 2. Analysis of Compounds in Kampo Medicines

### 2.1. Low-Molecular-Weight Compounds in Kampo Medicines, Crude Drugs, and Other Natural Products

Several Kampo medicine crude drugs [1,2,3] have been analyzed to determine the primary component influencing cellular or organ function. For example, using 3D-HPLC, Kishida et al. and Nakanishi et al. identified the components of the Goshajinkigan extract, including morroniside, (+)–catechin, loganin, paeoniflorin, penta-*O*-galloylglucose, benzoylmesaconine, cinnamic acid, isoacteoside, benzoylpaeoniflorin, cinnamaldehyde, 16-ketoalisol A, and paeonol [14,16]. Meanwhile, Hosogi et al. identified paeoniflorin, hesperidin, and glycyrrhizic acid as the chemical markers of Ninjin’yoeito extract [16]. Low-molecular-weight compounds are generally extracted using low-polar solvents, such as methanol [10,59], acetone [60], hexane [10,61], and ethyl acetate [61]. These solvents can be evaporated at 40–60 °C, facilitating the facile collection of low-molecular-weight compounds [59,60,61]. Miyano et al. prepared a water extract of Hangeshashinto and, subsequently, prepared a methanol extract fraction from the water extract, identifying baicalin, glycyrrhizic acid, and berberine [62]; however, this process included a freeze-drying step, which is inconvenient when evaporating low-polar solvents.

### 2.2. Proteins in Crude Drugs

Proteins in the crude drugs of Kampo medicines have not been thoroughly analyzed against low-molecular-weight compounds. Hence, we introduced previous studies that evaluated challenging proteins in crude drugs, including Yokuinin (*Coix lachryma-jobi* L. var. *Ma-yuen* Stapf.) and Mashinin (*Cannabis Fructus*). Some studies have analyzed low-molecular compounds or polysaccharides that can be extracted into a low-polarity solvent in Yokuinin and investigated their effects in vitro and in vivo [63,64,65,66,67,68,69]. However, Li et al. extracted the components in Yokuinin using high-polarity solvents, namely, 0.5 M sodium chloride aqueous, 70% ethanol aqueous, and 12.5 mM sodium borate buffer [18]; these four solvents contained albumin, globulin, prolamin, and glutelin, and their target was glutelin. Due to the high molecular weight of glutelin, Li et al. performed acid hydrolysis of the glutelin and characterized the glutelin peptides using gel filtration chromatography and reversed-phase HPLC (RP-HPLC). Although the low-molecular compounds in *Cannabis* used as crude drugs and commercial product resources for humans have been thoroughly investigated [70,71,72], their proteins have not. Hence, Liao et al. extracted proteins from *Fructus Cannabis* using water and analyzed them using Fourier transfer infrared (FT-IR) and ultraviolet spectrum (UV) spectroscopy [19]. The proteins were hydrolyzed to obtain various peptides, which were analyzed via liquid chromatography mass spectrometry (LC-MS).

## 3. Previous and Potential Technologies for the Identification and Quantification of Proteins in Kampo Medicines

High-molecular-weight compounds (>10 kDa), such as proteins and polysaccharides, should be extracted with high-polarity solvents, and samples were performed as the freeze-drying method for water removal [18,19,25]. Certain column chromatography protocols can separate and collect proteins from samples [22,23,24,25]. Moreover, silica gel normal phase, reverse normal phase, gel filtration, and ion exchange chromatography have been employed as liquid chromatography methods [25,73,74]. However, the low protein concentration in Kampo medicines may hinder their identification via liquid chromatography. Moreover, if samples undergo a freeze-dry treatment and are injected into the column, the separated proteins must be subjected to another round of freeze-drying [25], thus complicating the overall process. Meanwhile, silica gel normal phase and reverse phase chromatography are unsuitable for separating proteins due to their unstable stationary phases, which cannot be probed with high-molecular-weight compounds [73]. Although researchers can select gel filtration and ion exchange chromatography to separate and collect proteins, the solvent of the mobile phase must be highly polar [73,74]. Additionally, if the solvent used for analysis with gel filtration and ion exchange chromatography contains ions, such as sodium, the samples must be desalted.

Although researchers typically use the Bradford [75,76] or BCA methods [77,78], these require polypropylene tubes and 96-well microplates. When measuring protein concentrations, 100–1000 μL of a sample is required, comprising cell lysate/tissue lysate and Bradford or BCA reagents. However, the proteins in the water extract of Kampo medicines (or crude drugs) are often low and may be undetectable. When researchers investigate intracellular or tissue proteins in vitro or in vivo, Western blot with SDS-PAGE [22,26] and ELISA [27,28] are commonly used to identify or quantify the individual proteins (e.g., interleukin-1, tumor necrosis factor-α, and matrix metalloproteinase) [79,80,81]. The volume of samples and reagents used is 10–30 μL per SDS-PAGE well and 50–200 μL per ELISA well. Therefore, individual and rare proteins in Kampo medicines (or crude drugs) are not effectively identified or quantified using these methods.

Membrane chromatography can effectively separate and collect proteins [22,23,24,25]. The membrane is used as the stationary phase, while the mobile phase (e.g., liquid or gas) is vertically or parallelly flowed against the membrane. The samples can be continuously run until the collection is complete. If the protein concentration in the samples is low, high sample volumes in the liquid or gas phase can flow repeatedly against the membrane. These membranes primarily comprise cellulose acetate, cellulose/acrylic composite, chitin, chitosan, nylon, and PVDF [24]. Meanwhile, Ogino et al. developed a filter blot method with a PVDF membrane to analyze 3-nitrotyrosine (3-NT)-modified proteins in the atmosphere and compared the results with those obtained using HPLC-electrochemical detection (ECD) (HPLC-ECD) [37]. The 3-NT-modified proteins concentration determined via the filter blot method significantly correlated with that using the HPLC-ECD method (r = 0.809, *p* < 0.001). Moreover, Bickner et al. separated proteins with SDS-PAGE, transferred proteins onto PVDF membranes, and performed “on-PVDF membrane digestion.” They then identified proteins with ESI-MS analysis [38]. Although researchers generally perform “in-gel digestion” to identify proteins with ESI-MS or matrix-assisted laser desorption/ionization mass spectrometry (MALDI-MS) [26], “on-membrane digestion” is a high-level technology. Meanwhile, slot blot analysis is based on the principle of membrane chromatography with the sample flowing vertically against the membrane. Therefore, we postulate that slot blot analysis can identify and quantify rare proteins in Kampo medicines. Generally, nitrocellulose or PVDF membranes are selected for the slot blot analysis [25]. Although PVDF has been rarely reported, it offers good membrane-forming properties, thermal stability, chemical stability, and mechanical properties [33,34]. We consider that researchers have favored nitrocellulose membranes because their lysates of cells or tissues are deemed unsuitable for PVDF membranes. However, we have discovered a unique lysis buffer suitable for application with PVDF membranes [24,25].

## 4. Equipment, Characteristics, and Methodology of the Novel Slot Blot

### 4.1. Equipment

**Figure 1:**
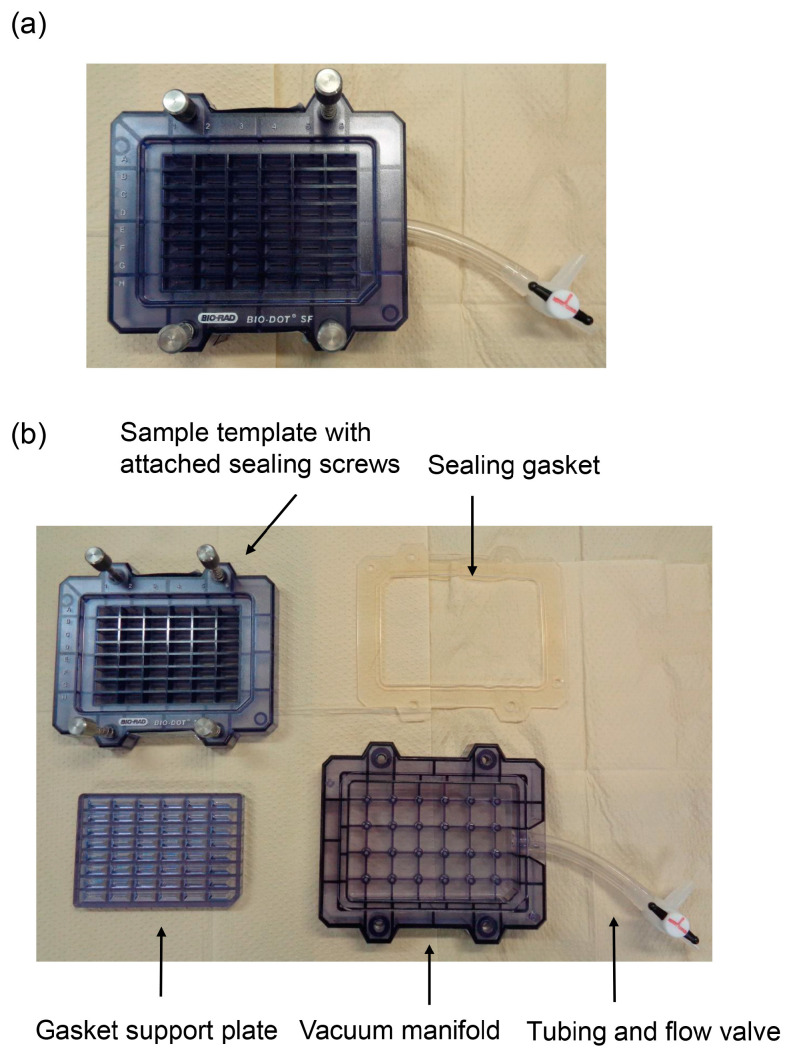
Bio-dot SF microfiltration apparatus (slot blot apparatus with 48 wells). (**a**) Assembly of the apparatus. (**b**) Disassembled apparatus.

The novel slot blot method was performed using a Bio-Dot SF Microfiltration Apparatus (Cat. no.: 170-6452; Bio-Rad Laboratories Inc., Hercules, CA, USA) with 48 wells (Figure 1).

### 4.2. PVDF Membrane

**Figure 2:**
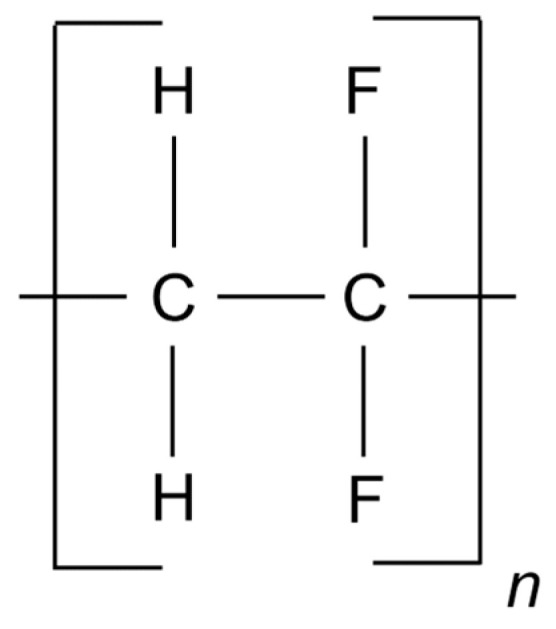
Chemical structure of the PVDF membrane. The “n” indicates that the structure repeated.

The novel slot blot method was performed using a PVDF membrane (Cat. no.: IPVH00010, pore size: 0.45 μm; Merck Millipore, Darmstadt, Germany). The chemical structure comprised carbon combined with hydrogen and fluorine atoms (Figure 2).

Nitrocellulose membranes, not PVDF membranes, are generally used to perform slot blot analysis on various proteins [82,83,84,85,86,87,88,89]. Although protein absorption and PVDF membrane durability are superior [33,34], researchers avoid performing slot blot analysis to identify or quantify proteins. PVDF membranes are commonly used for Western blot analysis [39,40] and can combine with C=O and N–H groups, rendering them superior for protein absorption [33]. However, the appropriate conditions for directly applying protein-containing samples onto a PVDF membrane have not been achieved. Given that electric current transports the proteins during Western blotting, proteins become transferred from the gel to the PVDF membrane. Therefore, the benefits of the PVDF membrane for protein absorption using slot blot can be demonstrated when a superior sample solution is used.

### 4.3. Lysis Buffer

For our novel slot blot, a custom lysis buffer was produced that differed from commonly used commercial lysis buffers [39,40]. First, tris-(hydroxymethyl)-aminomethane (Tris) (Cat. no.: 011-20095; Fujifilm Wako Pure Chemical, Osaka, Japan), urea (Cat. no.: 217-01215; Fujifilm Wako Pure Chemical), thiourea (Cat. no.: 201-17355; Fujifilm Wako Pure Chemical), and 3-[3-(cholamidopropyl)-dimethylammonio]-1-propanesulfonate (CHAPS) (Cat. no.: 347-04723; Dojindo Laboratories, Kumamoto, Japan) were dissolved in ultrapure water to prepare a solution of 30 mM Tris, 7 M urea, 2 M thiourea, and 4% CHAPS (Solution A, Table 1). Second, a protease inhibitor cocktail tablet (Complete Tablets EDTA-Free, EASY pack, Cat. no.: 04-693-132-001; Roche, Bavaria, Germany) was dissolved in ultrapure water (final volume: 2 mL, Solution B, Table 1 [39,40,90]). Finally, Solutions A and B were mixed (9:1) to form Solution C (Table 1), comprising 27 mM Tris, 6.3 M urea, 1.8 M thiourea, and 3.6% CHAPS. Solution C served as the lysis buffer for our assay [39,90,91,92,93,94,95,96]. To create Solution D, Solution B was added to the solution containing Tris, urea, thiourea, and CHAPS in ultrapure water (Table 1) [97,98,99,100].

Solution C was prepared following the method described in eight previous studies, and Solution D was prepared following four to quantify intracellular AGEs using the novel slot blot (Table 2). Although PVDF membranes have been previously used in slot blots to quantify proteins, the lysis buffer containing Tris, urea, thiourea, and CHAPS has not been used [101,102,103]. Gravel et al. used 4 M urea/Tris-buffered saline to quantify influenza type A viral hemagglutinin [103], whereas Papadaki et al. used 8 M urea and 0.1% SDS [104]; these lysis buffers are similar to ours. In contrast, Takino et al. employed a radioimmunoprecipitation (RIPA) buffer for their analysis of large sample concentrations (30 μg of proteins) with their slot blot analysis [39,40,102], whereas our novel method is suitable for samples with small amounts of protein (2.0 μg of proteins). Although RIPA buffer components (e.g., Triton-X) cause denaturation, they may inhibit protein probing onto the PVDF membrane. Papadaki et al. homogenized cardiac tissues with standard rigor buffer containing 1% Triton-X; they then removed the Triton-X and resuspended the pellet in a buffer containing 8 M urea and 0.1% SDS [104], revealing that Triton-X inhibited slot blot analysis.

**Figure 3:**
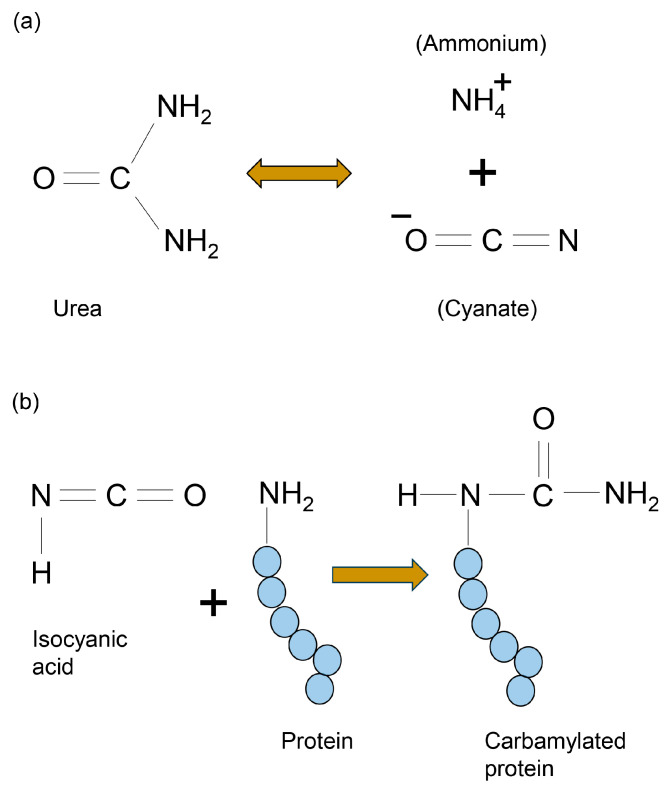
Mechanism of carbamylated protein formation with urea. (**a**) The reaction path of ammonium and isocyanate from urea. (**b**) Isocyanic acid attack on N-terminal lysine, arginine, and cysteine residues in protein.

Our ideal lysis buffer must promote protein denaturation and not inhibit PVDF membrane probing. When developing this novel slot blot assay, we prepared the lysis buffer based on those selected for two-dimensional electrophoresis (2-DE)-based protein division treatment [39,40]. Meanwhile, many studies have used 7 M urea and 2 M thiourea [105,106,107,108,109,110,111,112,113,114,115] with 2% [109], 3% [105,112], or 4% [106,107,108,110,111,113] CHAPS. Based on previous research [113,114], we hypothesized that our lysis buffer promotes protein probing on the PVDF membrane surface. According to McCarthy et al. and Herbert [113,114], urea, thiourea, and CHAPS can denature proteins by acting as chaotropic reagents and surfactants; these reagents disrupt hydrogen bonding and cause protein unfolding, exposing hydrophobic amino acid residues to the solution. CHAPS is combined with urea and thiourea to coat hydrophobic residues and improve solubility, and thiourea/urea combinations are widely used to exploit thiourea’s improved denaturing ability [113]. Furthermore, urea may be more important in inhibiting protein probing on the PVDF membrane. Urea reacts with ammonium and cyanate, with cyanate particularly adept at producing isocyanic acid that can subsequently react with *N*-terminal amino groups as well as lysine, arginine, and cysteine residues in proteins, producing carbamylated proteins (Figure 3) [114]. Given that protein C=O and N–H groups react with the PVDF membrane [34], carbamylation may promote protein adhesion. Furthermore, Tris has been used to stabilize the pH range of cell lysates at 8.5–8.8 [105,107]. Previous studies have used 30 mM [106,107] or 40 mM [105]. We determined the final concentration of urea, thiourea, CHAPS, and Tris in our lysis buffer (Solution C and Solution D, Table 1) based on their various concentrations in previous 2-DE studies.

We compared each slot blot analysis with different buffers and with nitrocellulose or PVDF membranes (Table 2).

### 4.4. Application of Standard and Sample Solutions and Vacuum with Water Aspirator

**Figure 4:**
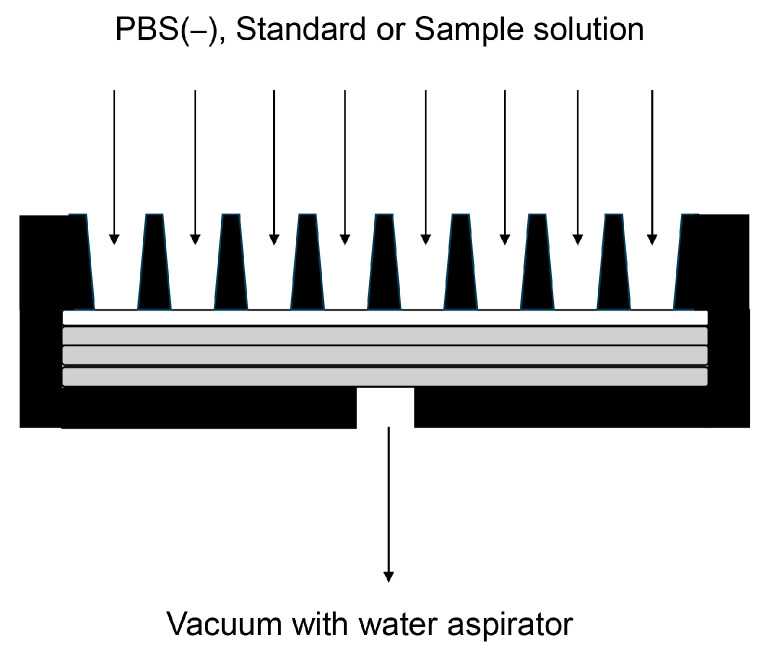
Slot blot apparatus with one PVDF membrane and three filter papers. Chambers are designed for PBS(–), standard and sample solution addition, and vacuum generation. A closed white rectangle represents the PVDF membrane. Closed gray rectangles represent filter papers.

Cell or tissue lysates were prepared with Solution C or Solution D (Table 2) [39,40,90,91,92,93,94,95,96,97,98,99,100]. The protein concentration of the samples was measured using the Bradford method, and equal amounts of cell or tissue lysate (e.g., 2, 4, and 10 μg of protein) were collected [39,40,91,92,93]. According to the Bio-Rad manufacturing protocol, 200–500 μL of solution should be applied to the membrane; hence, we added 200 μL of the standard or sample solution. Moreover, we diluted each sample with lysate buffer to ensure equal concentrations [40,91,92,93]. In our previous study, the volume of the cell or tissue lysate and additional lysis buffer was approximately 4–15 μL, and phosphate-buffered saline (PBS)(–) was added for a final volume of 200 μL. To denature the standard (e.g., AGE-modified protein), it was dissolved in lysis buffer and PBS(–) [39,40,91,92,93]. The PVDF membrane was activated with methanol before incubation in PBS(–). Three filter papers were then incubated in PBS(–) according to Bio-Rad’s protocol. The PVDF membrane and three filter papers were set in the slot blot apparatus (Figure 4).

Only the upper side of the PVDF membrane was exposed to air, and PBS(–), standard, and sample solutions were added from the top. The lower side of the PVDF membrane adhered to the filter paper containing the PBS(–). The water aspirator vacuum was applied from the lower side of the PVDF membrane (Figure 4).

**Figure 5:**
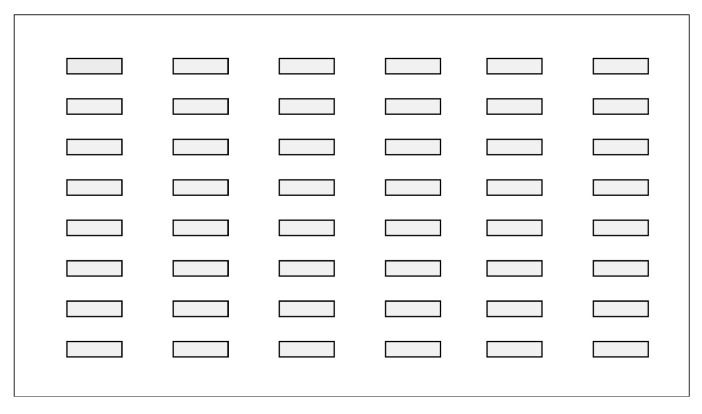
Wells in the slot blot for solution application in the slot blot apparatus. Closed gray squares represent slot lanes.

The PVDF membrane and filter papers were fixed in the apparatus, and 48 wells were created on the surface of the PVDF membrane (Figure 5). Subsequently, we quantified AGEs using the apparatus [39,90,91,92,93,94,95,96,97,98,99,100].

**Figure 6:**
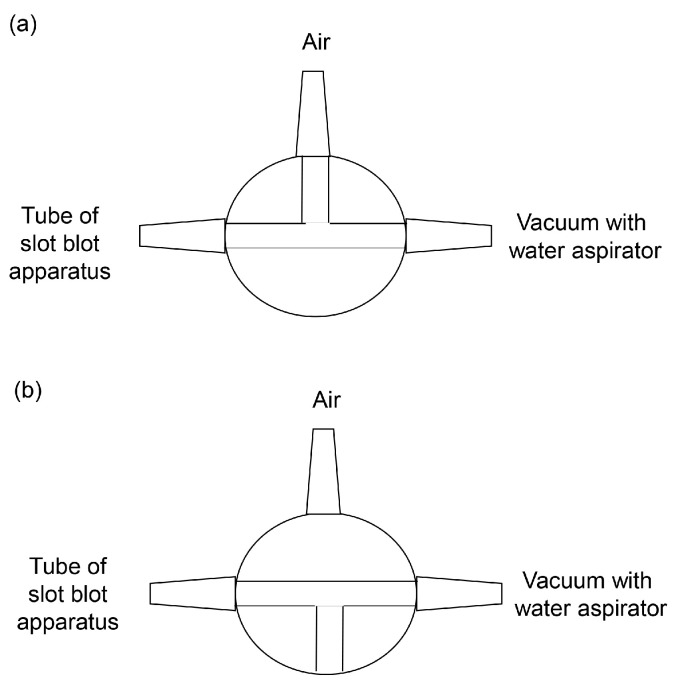
Valve between the slot blot apparatus tube and water aspirator vacuum. (**a**) The vacuuming with one valve opened against air. (**b**) The vacuuming with the valve closed against air.

Before applying the standard or sample solution, the PVDF membrane was washed with PBS(–) according to the Bio-Rad protocol. Accordingly, 100 μL of PBS(–) was added without water aspiration; subsequently, 200 μL of standard or sample solution was added with water aspiration, and one of the valves was opened against the air (Figure 6a). Although the water aspiration pressure was not specified, it was estimated. Water aspirator vacuuming was performed in the Kanazawa Medical University laboratory (Uchinada, Ishikawa, Japan). Water was collected from the water supply, managed with the storage tank, and resupplied to each laboratory. However, the water pressure remained constant, similar to that of a typical household or corporate water supply system in Uchinada. According to the Ministry of Health, Labour, and Welfare, the feed water pressure is 0.15–0.74 MPa in a typical Japanese household or corporation and 0.20–0.23 MPa in Uchinada. Therefore, all areas of Kanazawa Medical University’s water supply system have been adjusted so that their feed water pressure is 0.20–0.23 MPa. For a complete sample addition, we recommend vacuuming with water aspiration with the valve closed against the air (Figure 6b). After adding the standard or sample solution, 200 μL PBS(–) was applied and vacuumed with water aspiration with one valve opened (Figure 6a) and then closed (Figure 6b) against air. PBS(–) and other solutions were probed onto the PVDF membrane under a water aspirator vacuum, following Bio-Rad’s protocols.

### 4.5. Protein Quantification in Standard and Sample Solutions

We reported the quantification of certain GA-AGEs [39,90,91,92,93,94,95,96,98,99,100] and 1,5-anhydro-D-fructose AGEs [97] probed with primary antibody, secondary antibody, and chemiluminescent reagents. For example, 0–100 ng/well of standard AGE-modified proteins and approximately 10–20 ng/well (samples with 2.0 μg of protein were applied onto the PVDF membrane) of AGEs were detected [40,92].

## 5. Comparing the Novel Slot Blot with Other Slot Blots

Previous studies have reported statistical analysis on data obtained from slot blots performed with a nitrocellulose membrane and RIPA buffer (Table 3) [87,88]. However, those with a PVDF membrane and RIPA buffer did not provide data with error bars and the statistically significant difference [102]. Meanwhile, Gravel et al. presented their data obtained using a PVDF membrane with 4 M urea using error bars without the statistically significant difference [103]. In comparison, data obtained using the novel slot blot was presented with the information of both error bars and the statistically significant difference [39,90,91,92,93,94,95,96,97,98,99,100]. Moreover, we confirmed the suitability of Solutions C and D to promote PVDF membrane probing and facilitate statistical analysis, thus demonstrating the novelty of our assay. Although Papadaki et al. provided data with both error bars and the statistically significant difference [104], they homogenized cardiac tissues with standard rigor buffer containing 1% Triton-X in the first step of the assay and removed Triton-X in the second step. Ultimately, the pellet was resuspended in a buffer containing 8 M urea and 0.1% SDS [104]. Hence, although the data generated from our assay and that of Papadaki et al. were subjected to statistical analysis, our method requires fewer steps to prepare the lysate [39,40,104]. Furthermore, Solution C is suitable to homogenize cells and tissues. Although this was not confirmed for Solution D, we expect it will be as effective as Solution C.

## 6. Potential for Identifying and Quantifying Various Rare Proteins in Kampo Medicines Using the Novel Slot Blot Method

**Figure 7:**
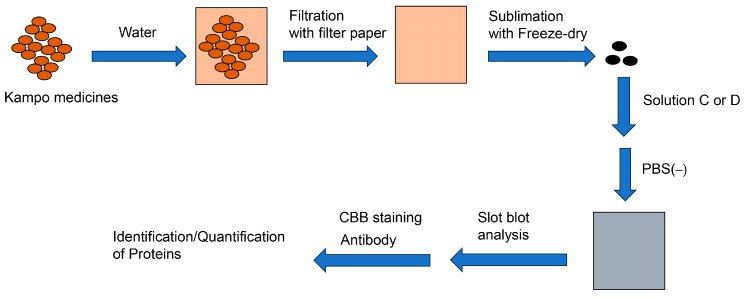
Potential for identifying and quantifying proteins in Kampo medicines. Closed brown circles: Kampo medicine; pale brown square: water extract of Kampo medicine; black circles: pellet samples from the water extract; closed gray square: solution C or D/PBS(–) in which the pellet samples are dissolved.

Our novel slot blot method can be used to identify and quantify proteins in Kampo medicines. Compared with test tubes and 96-well microplates, PVDF membrane filtration exhibited particularly good performance. Although the sample volume applied in studies using test tubes and 96-well microplates is typically limited, the slot blot analysis continued until the PVDF membrane became clogged (Figure 4). Hence, one of the slot blot’s distinguishing features is that the Kampo medicine extract can be repeatedly dropped onto the PVDF membrane and vacuumed with a water aspirator. Kampo medicines can be extracted with water and collected by removing the water using the freeze-drying method. These samples can be redissolved in Solution C or Solution D (Table 1) and then added to the appropriate PBS(–) solution. Proteins then accumulate on the PVDF membrane as the sample is applied repeatedly (Figure 4 and Figure 5). The accumulation of proteins on the PVDF membrane can then be analyzed using Coomassie Brilliant Blue (CBB) staining (Figure 7), which stains proteins in WB gels [26] and PVDF membranes [116,117,118,119]. When WB analysis is performed, samples containing 10–30 μg of protein [26,39,95] are applied to the gel chambers and transferred to the PVDF membrane. When we examined AGEs in kidney tissue, we used a large sample (30 μg of protein) [93]. Researchers can quantify proteins using CBB and our slot blot analysis using a standard curve with 0–100 ng of AGE-modified proteins; in this way, 10–20 ng of AGEs in 2.0 μg of a protein sample can be quantified [92,95,96]. Additionally, this slot blot method may help identify and quantify individual proteins using antibody-based methods such as ELISA (Figure 7) [79,80,81]. Although our method has risks, such as the binding of polysaccharides to PVDF membranes [120,121], proteins treated with Solution C or D show robust adhesion to the membrane, which could prove advantageous. Bickner et al. identified various proteins probed onto the PVDF membrane using the “on-PVDF membrane digestion” treatment and ESI-MS analysis. The proteins on the membrane were then identified and quantified using the slot blot and ESI-MS/MALDI-MS analysis [38]. However, we consider this strategy to be more challenging than that described by Bickner et al., who performed WB to separate proteins, transferred the proteins onto the PVDF membrane from the gel, and separated them into six groups (the PVDF membrane was cut into six membranes). In contrast, when the slot blot analysis is performed, the proteins are within one area of the PVDF membrane. This is not beneficial for analysis with ESI-MS.

This study is limited by the absence of confirmatory identification and quantification of the proteins in Kampo medicines using the novel slot blot method. However, we consider that they are able to be detected because AGE-modified proteins in cells and tissue lysates were previously quantified with our slot blot [39,90,91,92,93,94,95,96,97,98,99,100]. Moreover, because the proteins in Kampo medicines should be extracted with high-polarity solvents such as water, any proteins that are not soluble in these solvents will not be detected. Also, we have not confirmed that whole proteins were extracted from Kampo medicines, which requires further verification.

## 7. Conclusions

Although analysis of rare proteins in Kampo medicines has proven challenging for conventional technology such as column chromatography, WB, and ELISA, methods based on the principle of membrane chromatography, such as slot blot, are effective. We consider that the slot blot analysis is suitable for identifying and quantifying proteins in Kampo medicines because this strategy allows samples to flow continuously without limitation against the membrane. Furthermore, we consider that our novel slot blot, comprising a PVDF membrane and specific lysis buffer, is most suitable as it provides data that show both error bars and the statistically significant difference compared with that produced by other similar assays, and our protocol is simpler, with fewer steps.

## Figures and Tables

**Table 1:** Solutions used to prepare the lysis buffer [39,40,90,91,92,93,94,95,96,97,98,99,100].

Solution A	Solution B	Solution C	Solution D
30 mM Tris 7 M Urea 2 M Thiourea 4% CHAPS (Ultrapure water) (pH 8.5)	1 Protease inhibitor cocktail tablet/2 mL (ultrapure water)	27 mM Tris 6.3 M Urea 1.8 M Thiourea 3.6% CHAPS 10% Solution B (Ultrapure water) (pH 8.5)	30 mM Tris 7 M Urea 2 M Thiourea 4% CHAPS 4% Solution B (Ultrapure water) (pH 8.5)

**Table 2:** List of references used for preparing Solutions C and D.

Solution	References
C	[39,90,91,92,93,94,95,96]
D	[97,98,99,100]

**Table 3:** Experimental conditions for slot blot and data analysis.

No.	Sample	Membrane Type	Lysis Buffer	Error Bars	Statistically Significant Difference	References
1	Cell lysate	Nitrocellulose	RIPA	Yes	Yes	[87,88]
2	Cell lysate	PVDF	RIPA	No	No	[102]
3	Protein in virus	PVDF	4 M Urea	Yes	No	[103]
4	Cell lysate	PVDF	Solution C	Yes	Yes	[39,90,92,94,95,96]
5	Tissue lysate	PVDF	Solution C	Yes	Yes	[91,93]
6	Cell lysate	PVDF	Solution D	Yes	Yes	[97,98,99,100]
7	Tissue lysate	PVDF	8 M Urea, 0.1%SDS	Yes	Yes	[104]

## Data Availability

The data presented in this study are available in the article.

## Abbreviations

AGEs	Advanced glycation end products
CHAPS	3-[(3-cholamidopropyl)-dimethylammonio]-1-propane sulfonate
PBS	Phosphate-buffered saline
PVDF	Polyvinylidene difluoride
Tris	Tris-(hydroxymethyl)-aminomethane
LC	Liquid chromatography
